# Transcriptional Regulation and Adaptation to a High-Fiber Environment in *Bacillus subtilis* HH2 Isolated from Feces of the Giant Panda

**DOI:** 10.1371/journal.pone.0116935

**Published:** 2015-02-06

**Authors:** Ziyao Zhou, Xiaoxiao Zhou, Jin Li, Zhijun Zhong, Wei Li, Xuehan Liu, Furui Liu, Huaiyi Su, Yongjiu Luo, Wuyang Gu, Chengdong Wang, Hemin Zhang, Desheng Li, Tingmei He, Hualin Fu, Suizhong Cao, Jinjiang Shi, Guangneng Peng

**Affiliations:** 1 The Key Laboratory of Animal Disease and Human Health of Sichuan Province, College of Veterinary Medicine, Sichuan Agricultural University, Ya'an, 625014, PR China; 2 Chengdu Center for Animal Disease Prevention and Control, Chengdu, 610041, PR China; 3 Ya'an Bifengxia Base, China Conservation and Research Center for the Giant Panda, Ya'an, 625007, PR China; Cornell University, UNITED STATES

## Abstract

In the giant panda, adaptation to a high-fiber environment is a first step for the adequate functioning of intestinal bacteria, as the high cellulose content of the gut due to the panda's vegetarian appetite results in a harsh environment. As an excellent producer of several enzymes and vitamins, *Bacillus subtilis* imparts various advantages to animals. In our previous study, we determined that several strains of *B. subtilis* isolated from pandas exhibited good cellulose decomposition ability, and we hypothesized that this bacterial species can survive in and adapt well to a high-fiber environment. To evaluate this hypothesis, we employed RNA-Seq technology to analyze the differentially expressed genes of the selected strain *B. subtilis* HH2, which demonstrates significant cellulose hydrolysis of different carbon sources (cellulose and glucose). In addition, we used bioinformatics software and resources to analyze the functions and pathways of differentially expressed genes. Interestingly, comparison of the cellulose and glucose groups revealed that the up-regulated genes were involved in amino acid and lipid metabolism or transmembrane transport, both of which are involved in cellulose utilization. Conversely, the down-regulated genes were involved in non-essential functions for bacterial life, such as toxin and bacteriocin secretion, possibly to conserve energy for environmental adaptation. The results indicate that *B. subtilis* HH2 triggered a series of adaptive mechanisms at the transcriptional level, which suggests that this bacterium could act as a probiotic for pandas fed a high-fiber diet, despite the fact that cellulose is not a very suitable carbon source for this bacterial species. In this study, we present a model to understand the dynamic organization of and interactions between various functional and regulatory networks for unicellular organisms in a high-fiber environment.

## Introduction

The intestinal microbiota, of which a major component is bacteria, greatly contributes to host nutrition, metabolism, immunity and other characteristics [1–3]. However, the gut environment is not an ideal location for intestinal bacteria because most growth and reproduction genes are inhibited [4]. For the giant panda (*Ailuropoda melanoleuca*), one of the most highly endangered mammals, with only 2,500 to 3,000 individuals survived in western China [5], a high-fiber vegetarian diet combined with a carnivore-like gastrointestinal system results in a harsh gut environment, as soft bamboo shoots and stems are the major food sources of the panda but this animal does not possess a rumen for fermentation [6,7]. Thus, adaptation to a high-fiber environment is the first step for the intestinal microbiota of the giant panda to act as a probiotic. Although several recent studies have established a framework for the community composition and functions of the panda intestinal microbiota via metagenomics [8–10], it is not yet well understood how a single organism can survive and adapt in the high-fiber gut environment of the giant panda.

As a vital bacterial species of the mammalian intestinal microbiome, *Bacillus subtilis* imparts various advantages to animals due to its excellent ability to produce several enzymes and vitamins [11,12]. In our previous study [13,14], we determined that several *B*. *subtilis* strains isolated from pandas demonstrated good cellulose decomposition capability as well as other contributions; thus, we speculated that this bacterial species could help the panda in several ways, including aiding in the digestion of bamboo in a high-fiber intestinal environment. Previously, glucose had been recognized as the preferred carbon source for microbes [15], while many bacterial species had been shown to not be able to use cellulose. Our previous results proved that *B*. *subtilis* had ability to survive in a high-fiber environment, but the mechanism is still unknown.

The literature has shown that the adaptation of *B*. *subtilis* in different environments occurs mainly through transcriptional regulation [16]. The levels of most bacterial stress adaptation molecules, such as the Hfq protein [17], small RNAs [18] and 16S rRNA [19], are determined at the transcript level. Therefore, we employed RNA-Seq technology to compare the differentially expressed genes (DEGs) of the selected *B*. *subtilis* HH2 strain when using cellulose and glucose as primary carbon sources, and we then analyzed the functions and pathways of the DEGs. In this study, we revealed major transcriptional reconfigurations in response to cellulose adaptation as well as certain coordinated changes in the abundance of *B*. *subtilis* HH2. We then produced a model to understand the dynamic organization of the interactions between various functional and regulatory networks of unicellular organisms in the giant panda intestine.

## Materials and Methods

### Bacterial strain and cultivation conditions

Glucose medium was modified from a previous study [20]: 70 mmol K_2_HPO_4_, 30 mmol KH_2_PO_4_, 25 mmol (NH_4_)_2_SO_4_, 0.5 mmol MgSO_4_, 10 μmol MnSO_4_, 22 mg ferric ammonium citrate, 8 g potassium glutamate, 6 g potassium succinate, 1% glucose, 0.5 mmol CaCl_2_, 5 μmol MnCl_2_, and 1000 mL of ddH_2_O at pH = 7.2. The cellulose medium was formulated in the same way as the glucose medium, except that the main carbon source was 1% sodium carboxymethylcellulose instead of 1% glucose.

*B*. *subtilis* HH2 from fresh feces that was collected from healthy pandas at the Ya'an Bifengxia Base of the China Conservation and Research Center for the Giant Panda (CCRCGP) and placed in sterile sampling bags in our previous study was isolated and identified. This strain has a good ability to digest cellulose; the diameter of its cellulose hydrolysis halo was 28.00±0.44 mm (S1 Fig.). The strain was grown in 100 mL of LB medium at 37°C in a shaker at 150 rpm for 24 h. After cultivation, 1% of the cells was inoculated into glucose and cellulose media and grown at 37°C in a shaker at 150 rpm until OD_600_~1.

### RNA isolation and preparation

Total RNA was extracted using the hot phenol method [21]. In brief, cell pellets were resuspended and washed once in Buffer A (50 mM sodium acetate and 10 mM EDTA, pH = 5.2). After collecting the cells by centrifugation, the pellets were resuspended in Buffer A containing 1% SDS and immediately added to hot phenol. After incubation at 65°C for 5 minutes followed by centrifugation for 10 minutes at 4°C, the RNA-containing supernatants were transferred to a new tube for ethanol precipitation, washed and then dissolved in DEPC-treated water. The RNA was further purified with two phenol-chloroform treatments and then treated with RQ1 DNase (Promega) to remove DNA. The quality and quantity of the purified RNA were determined by measuring the absorbance at 260 nm/280 nm (A260/A280) using Smartspec Plus (BioRad). The integrity of the RNA was further verified by 1.5% agarose gel electrophoresis.

### cDNA library construction and sequencing

Ribosomal RNAs were removed from the RNA samples (10 μg) using a RiboMinus rRNA depletion kit (Ambion), and the resulting samples were used to prepare directional RNA-Seq libraries [22, 23]. The purified mRNAs were then iron-fragmented at 95°C followed by end repair and 5' adaptor ligation. Then, reverse transcription was performed using RT primers containing a 3' adaptor sequence and a randomized hexamer. The cDNAs were purified and amplified, and all 200-500-bp PCR products were purified, quantified and stored at -80°C until they were used for sequencing.

For high-throughput sequencing, the libraries were prepared following the manufacturer's instructions, and the Illumina GAIIx system was used to collect data from 80-nt single-end sequencing (ABlife Inc.; Wuhan, China).

### Alignment of reads to the genome

After obtaining the sequencing data, the raw data were screened, which included removal of two-N-containing reads, removal of the sequence adaptor, and identification of clean reads with lengths of more than 16 nt after removal of low-quality values. Because the 16S rRNA gene of *B*. *subtilis* HH2 has maximal homology with Bacillus_subtilis_PY79 (CP006881.1) when compared with all *B*. *subtilis* genomes in the NCBI database (before November 11, 2013), we decided to use Bacillus_subtilis_PY79 as the reference genome for *B*. *subtilis* HH2. We utilized Tophat [24] with 2-nt mismatches to align our sequencing data to the reference genome of *B*. *subtilis* HH2 (ftp://ftp.ncbi.nlm.nih.gov/genomes/Bacteria/Bacillus_subtilis_PY79_uid229877/). Using the RPKMs (reads per kilobase of a gene per million reads) [25], we eliminated the deviations due to the lengths of different genes.

### Analysis of differentially expressed genes

To perform differential gene expression analysis, we applied the software edgeR [26], which is specifically used to analyze the differential expression of genes using RNA-Seq data. To determine whether a gene was differentially expressed, the analysis results were based on the fold change (FC≥2 or FC≤-2) and P-value (P≤0.01). To predict gene function and calculate the functional category distribution frequency, KEGG and Gene ontology (GO) analyses were employed using DAVID bioinformatics resources [27].

## Results and Analysis

### Cellulose is not a highly suitable carbon source for *B*. *subtilis* HH2

**Fig 1:**
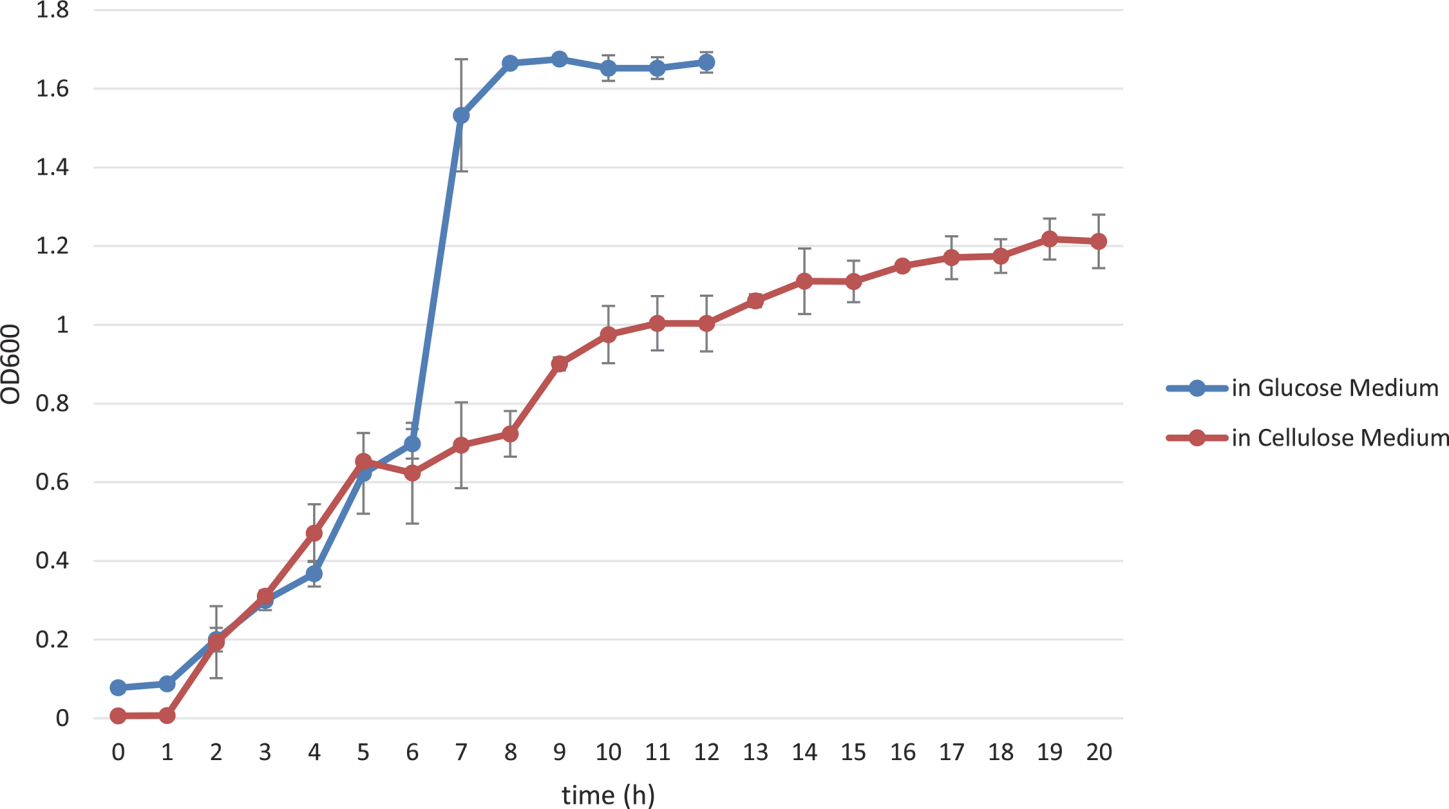
The growth curves of *B*. *subtilis* HH2 exposed to different carbon sources. *B*. *subtilis* HH2 was cultured in cellulose or glucose medium following 1% inoculation at 37°C in a shaker at 150 rpm; the OD_600_ was measured every hour. Each graph represents the mean of three independent biological replicates grown on three different days. The error bars represent the standard deviations (SDs) of the optical density at each time point.

**Fig 2:**
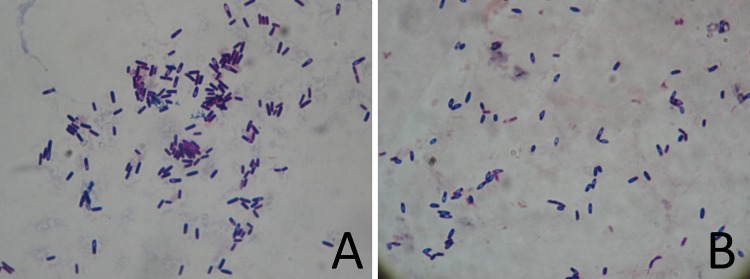
Bacteria under the light microscope. (A) *B*. *subtilis* HH2 was cultured in glucose medium until OD_600_~1. (B) *B*. *subtilis* HH2 was cultured in cellulose medium until OD_600_~1.

*B*. *subtilis* HH2 was first cultured in media supplemented with cellulose or glucose as the primary carbon source. We found that the growth of HH2 was significantly inhibited in the cellulose medium compared with the glucose medium (Fig. 1). In addition, when the bacterial culture reached OD_600_~1, we observed both the cellulose- and glucose-grown bacterial samples under a light microscope. Bacterial spores were more apparent in the cellulose medium than in the glucose medium (Fig. 2), which indicated that cellulose is not a suitable energy source for this bacterial strain.

### Profile of the RNA-Seq data

After cultivation, we extracted total RNA from the bacteria to analyze the transcriptional regulation in the different carbon environments via next generation sequencing (NGS). We obtained 20,231,277 and 24,807,479 fragments from the cellulose and glucose groups, respectively. After screening, 14,733,930 and 20,066,967 clean reads were generated from the two groups, respectively. We then analyzed whether these reads matched the reported reference genome in the GenBank database using blastn (E-value≤1e-5). We found that more than 75% of the reads could be mapped to the reference genome, of which approximately 50% were uniquely mapped reads, representing a combined sequence coverage of 250X (Table 1). In this study, the detected expressed genes (mapped reads number≥10) comprised 81.67% (3,279/4,278) of the reads for the cellulose group and 92.69% (3,779/4,278) of the reads for the glucose group. The total number of detected genes in the two samples was 4,134, which accounted for 96.63% of the *B*. *subtilis* genes, indicating that the gene detection in this study largely reached saturation.

**Table 1:** Mapping of clean reads in the *B*. *subtilis* genome.

Sample	Input reads	Total mapped	Unique mapped	Multiple mapped
Cellulose	14,733,930	11,293,808 (76.65%)	5,314,734 (47.06%)	5,979,074 (52.94%)
Glucose	20,066,967	16,316,254 (81.31%)	10,742,645 (65.84%)	5,573,609 (34.16%)

The correlation coefficient (R^2^) for the expression of the same gene in the two samples was 0.739, which demonstrated that parts of genes exhibited changes in expression levels. When comparing the cellulose group with the glucose group, the number of significantly down-regulated genes (by more than 10 times) was 164, which was significantly greater than the number of up-regulated genes (23 genes), illustrating that these organisms may significantly reduce the expression of some genes and lose partial function as a way to cope with the impacts of an extreme environment [28]. According to GO functional analysis, 23 gene-function clusters were enriched among the up-regulated genes when comparing the cellulose group to the glucose group, and 22 clusters among the down-regulated genes were enriched (S1 Table). Interestingly, among the clusters with the top 10 enrichment scores, the functions of the up-regulated genes (clusters) were mainly associated with cellulose utilization, whereas most of the down-regulated genes were associated with non-essential functions for bacterial life, indicating a reduction in energy consumption to permit environmental adaptation (Table 2).

**Table 2:** GO term analysis of DEGs (top 10 enrichment scores).

Differentially expressed gene cluster	Description	Enrichment Score
Down-regulated Cluster 1	Toxin, peptide, antibiotic and bacteriocin metabolic processes	3.96
Down-regulated Cluster 2	Chemotaxis, taxis and locomotor behavior	2.59
Down-regulated Cluster 3	Flagellar assembly and motility	2.29
Down-regulated Cluster 4	Membrane and transmembrane	2.00
Down-regulated Cluster 5	Flagellar assembly and bacterial flagellum protein export	1.98
Down-regulated Cluster 6	Cellular macromolecular complex assembly	1.28
Down-regulated Cluster 7	Anion transport	1.14
Down-regulated Cluster 8	ABC transporters	0.79
Down-regulated Cluster 9	Cell wall biogenesis/degradation	0.78
Down-regulated Cluster 10	Metal-binding	0.68
Up-regulated Cluster 1	Amino acid metabolism	1.44
Up-regulated Cluster 2	Transmembrane	1.23
Up-regulated Cluster 3	Carbohydrate transport	1.23
Up-regulated Cluster 4	Oxidoreductase and electron carrier activity	1.02
Up-regulated Cluster 5	Cell wall macromolecule catabolic processes	0.74
Up-regulated Cluster 6	Protein transport and localization	0.72
Up-regulated Cluster 7	Calcium ion binding and substrate binding	0.56
Up-regulated Cluster 8	Aminoglycan and polysaccharide catabolic processes	0.55
Up-regulated Cluster 9	Amino acid transmembrane transporter activity	0.52
Up-regulated Cluster 10	Sporulation	0.50

### Carbon metabolism gene expression is significantly altered in both groups

Generally, carbon metabolism-related gene cluster expression is altered in bacteria in conjunction with changes in nutritional factors. We found that the most significantly up-regulated clusters in the cellulose group were involved in the metabolism of amino acids, lipids, galactose, and ketone bodies, which indicates that when a directly used carbon source (usually beta-D-glucose) in the environment is limiting, an organism may consume other intracellular substances for supplementation. Cellulase component genes, such as the beta-glucanase gene U712_19750, were significantly highly expressed in the cellulose group compared to the glucose group. Similar observations were noted for U712_19485 and U712_19495, genes that encode two subunits of the lichenin-specific phosphotransferase enzyme, which has been confirmed to be involved in the digestion of cellobiose, a secondary product of cellulose catalysis [29]. To further utilize cellobiose, various forms of glucosidase (such as U712_19480 and U712_03600) are also up-regulated to create the final product beta-D-glucose.

In contrast, the expression of glucose metabolism-related genes was significantly decreased in the cellulose group compared to the glucose group, particularly at the start of glucose metabolism. In addition, the expression of the UDP-glucose 6-dehydrogenase *tuaD* (U712_17840), a necessary enzyme in the pentose phosphate pathway [30], was decreased by more than 16-fold. Furthermore, at the beginning of glucose metabolism, the expression of gluconate kinase (U712_20270) was decreased 9-fold in the cellulose group compared to the glucose group; and at the same time, the downstream production of glucose 6-phosphate, which is the substrate for the pentose phosphate pathway, was decreased [31]. S2 Table shows the differential expression of various genes that are important for carbon metabolism in the two samples.

### The expression of most non-essential genes is decreased in the cellulose group to reduce energy consumption

**Fig 3:**
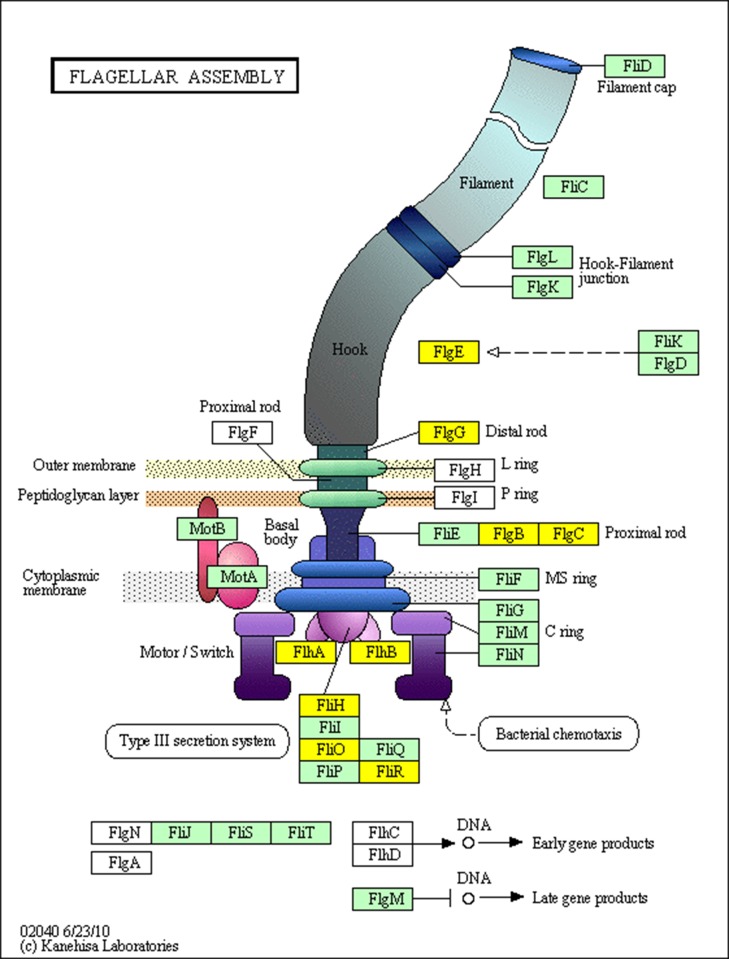
KEGG analysis of flagellar assembly (bsu02040) in *B*. *subtilis*. Yellow boxes indicate significantly down-regulated genes in the cellulose group, and gray boxes indicate up-regulated genes (none in this figure). Green indicates a group of proteins.

Apart from glucose metabolism genes, the down-regulated genes in the cellulose group were involved in chemotaxis, secretion of toxins and bacteriocins, motility, polymer compound assembly, and complex protein assembly. The expression of the subtilosin-A assembly gene U712_18825 was sharply decreased by ~187-fold. In addition to the decreased expression of genes involved in the assembly of complex substances, the down-regulation of certain non-essential genes reduced the energy consumption of the cell. For example, multiple vital genes in the flagellar assembly gene cluster (Fig. 3) were significantly decreased in the cellulose group. In addition, the expression of nearly every structural protein gene within the type III secretion system (T3SS) of the flagellar assembly gene cluster was down-regulated. Furthermore, the expression of a series of flagellar genes was decreased, indicating that motility and infection ability were decreased during nutritional deficiency. Given the inhibition of various non-essential metabolic genes as well as of genes involved in non-essential functions in the cellulose group, it is apparent that *B*. *subtilis* HH2 triggers a series of mechanisms that conserve energy to be used for adaptation to a high-fiber environment.

### Two-component systems (TCSs) and ATP-binding cassette (ABC) transporters are important regulatory systems for high-fiber environment adaption

When bacteria experience stress, they signal to the organism that an appropriate physiological response is required. TCSs and ABC transporters have been proposed to be units that participate in the common physiological process of signal transduction and substance transport [32,33]. In *B*. *subtilis*, the *yts*, *yvc* and *yxd* gene clusters encode an ABC transporter from sub-family 9 and a coupled TCS from the *OmpR* family [32], and the transcription factor of this system can activate transcription by binding DNA and interacting with RNA polymerase [34]. We found that the expression of each gene in the three clusters, as well as those of the corresponding ABC transporters, was decreased by more than half in our simulated intestinal environment. Given the involved signaling cascade, the reduced expression of the *OmpR* family may regulate the inhibition of many genes. Furthermore, the expression of several zinc-binding lipoproteins and zinc metalloproteases was decreased in the cellulose group, which could negatively affect the expression of many enzymes, storage proteins, transcription factors, and proteins involved in replication [35]. Therefore, TCSs and ABC transporters both regulate energy conservation in response to a high-fiber environment.

Conversely, TCSs and ABC transporters are also widely involved in nutrient absorption. In *B*. *subtilis*, ATP-driven uptake systems prefer primary carbon and energy sources [36]. Several kinases in TCS pathways involved in the metabolism of some amino acids were observed to be up-regulated in cellulose medium, e.g., *GlnT* (U712_01235) and *YesM* (U712_03510), which indicates changes in the expression of carbon metabolism genes. Several high-affinity ABC transporters for different sugars catalyze the transport of sugar-oligomers to an even greater degree than peptide transporters, which permits organisms to thrive in nutrient-poor environments [37,38]. In this study, the expression of many oligosaccharide and polyol transporters involved in cellulose hydrolyzate transport were increased in the cellulose group; for example, the expression of the gene U712_03520, which encodes the putative ABC transporter substrate-binding protein *yesO*, was increased 11.26-fold compared to the glucose group. It is likely that the expression of many peptide- and calcium-binding ABC transporter protein genes (such as U712_06760, U712_06745 and U712_08240) was enhanced to improve the transport of cellulose hydrolyzates.

### Structural membrane and transporter proteins are key factors for adaptation to stress

As the first organelle to experience pressure and the key organelle for adaptation to a harsh environment, the cell membrane makes vital contributions to combat the effects of environmental aggression, such as initiating the secretion of several proteins and exchanging intracellular and extracellular substances [38]. In this study, membrane and transmembrane genes were enriched in both the up- and down-regulated gene clusters according to GO analysis (S1 Table); however, on the individual level, the specifics of this enrichment were completely different. The up-regulated clusters primarily consisted of transporter proteins, including carbohydrate transporters, sugar transporters, and symporters, indicating that cellulose hydrolyzate may require a greater amount of energy to be delivered into the cell. In contrast, structural proteins, such as plasma membrane, cell membrane, and intrinsic membrane proteins, were primarily down-regulated. The down-regulation of structural membrane genes increases the permeability of the cell membrane, allowing the easy transport of cellulose hydrolyzates into the bacteria. Therefore, with a series of adjustments, the cell membrane could become more adapted to cellulose medium.

### Sporulation is a last-resort response to pressure and is suppressed until alternative responses prove inadequate

When alternative responses prove inadequate to relieve stress, sporulation is the fate chosen by most *Bacillus* species [39]. We observed increased sporulation in the cellulose group, both under the light microscope (Fig. 1) and at the transcriptional level (U712_12580, encoding the sporulation inhibitor *Sda*, was decreased more than 16-fold). However, among the seven stages (0-VI) of sporulation protein genes (clusters), which are proteins that determine the sporulation form, only stage II and stage III sporulation proteins were observed to be significantly up-regulated in the cellulose group. In contrast, the expression of proteins involved in the five other stages of sporulation was not greatly changed. Therefore, cellulose remains an acceptable carbon source for *B*. *subtilis* HH2, and this strain can adapt well to a high-fiber environment through a series of alterations in transcriptional regulation.

### A regulatory model of *B*. *subtilis* HH2 in a high-fiber environment

As described above, we revealed major transcriptional reconfigurations in response to cellulose adaptation as well as certain coordinated changes in the abundance of *B*. *subtilis* HH2 in a high-fiber environment. To summarize these adaptation mechanisms, we propose a regulatory model of *B*. *subtilis* HH2 for high-fiber environmental adaptation and cellulose digestion. In this model, utilization of and adaptation to cellulose require at least four functional classes of proteins, including (i) membrane proteins and membrane-associated signal channels, (ii) enzymes that catalyze cellulose hydrolysis, (iii) proteins encoded by operons that decrease cellular energy and nutrient consumption, and (iv) proteins involved in sporulation. The cellular degradation of and adaptation to cellulose consist of five steps. First, when bacteria are grown on a medium with cellulose as a primary carbon source, ion channel-coupled receptors in the cell membrane are stimulated by cellulose and send signals to the associated transduction systems. As a result, cellulase components are expressed, secreted, assembled and transported to the cell surface, which hydrolyzes the cellulose. Cellobiose and glucan, both products of cellulose hydrolyzation, are transported into the cell through channels for hydrolyzates and ABC transporters for further utilization. Simultaneously, upon pressure signals, bacteria partially reduce the synthesis of non-essential proteins to save energy. The expression of sporulation genes also partially increases to address potential continuing harsh pressures.

## Discussion

*B*. *subtilis* is widely used as a probiotic and food additive with mammalian applications due to its excellent ability to secrete a variety of antimicrobial substances that maintain the intestinal microflora balance [40] and improve the digestibility of foraged foods in the gastrointestinal tract [41]. A number of studies have examined *B*. *subtilis* resistance [16,19], but there is still a relative dearth of research on environmental cellulose adaptation mechanisms. A high-fiber environment is most characteristic of the herbivorous animal gut environment, particularly for the giant panda, which has no rumen for the fermentation of vegetation. In addition to the genomic potential of the intestinal microbiota, understanding bacterial adaptation mechanisms for a cellulose environment will help us to better clarify the interactions between intestinal bacteria and their panda host.

Through phenotype experiment of *B*. *subtilis* HH2 isolated from pandas and grown on different carbon sources, we demonstrated that cellulose is not a very suitable carbon source for *B*. *subtilis* HH2. However based on our transcriptional pathway analysis, we found that this bacterium can adapt well via a series of regulatory networks and that the differentially expressed genes clustered into two main categories: cellulose utilization and high-fiber environment adaptation. For cellulose utilization, strain HH2 not only increased the expression of cellulase but also of a series of enzyme components that hydrolyze cellulose, as well as some ABC transporters, which served as support. Whereas it has been shown that organisms can selectively express some genes highly for stress adaptation [39,42], in this study, it was observed that many genes (clusters), such as several protein kinases, were up-regulated but that the expression of most non-essential genes were down-regulated to conserve energy.

Interestingly, the expression of the Hfq protein (U712_09100), several sporulation kinases and genes of proteins involved in sporulation was decreased in the cellulose group. The Hfq protein is an RNA-binding protein associated with small regulatory RNAs (sRNAs) and has many functions in pressure adaptation [17]. Sporulation is the final adaptation by the genus *Bacillus* to relieve stress, and the decrease in the expression of these genes indicates that cellulose may be an acceptable carbon source for *B*. *subtilis* HH2.

As an intestinal probiotic, *B*. *subtilis* HH2’s cellulose utilization ability could aid pandas in digesting bamboo. The bacterial metabolite substances, such as polypeptides and lipids, which could be digested by the host were produced in cellulose decomposing. In addition to its nutritional effects, HH2 also contributes to maintaining the intestinal microflora balance in the host, since the substances for antimicrobial effect and immune stimulation were continuous producing in a high cellulose environment. Based on the gene expression data, we found that although HH2 decreasing some antimicrobial peptides expression in the cellulose medium compared to that in glucose medium may reduce its antimicrobial functions, most of bacitracin expression could still be detected in the cellulose group. Such as flagellin (U712_17730) and a series of surfactin components, which are involved in immune stimulation and resistance to pathogen colonization [40], were detected more than 2,000 reads. Thus, we believe that *B*. *subtilis* HH2 can still exert most probiotic functions both in nutritional and antimicrobial effects in a high-fiber environment within animals.

In summary, this study revealed major changes in transcriptional regulations in response to cellulose adaptation of *B*. *subtilis* HH2 on different carbon sources, as detected by RNA-Seq. These results demonstrate that this bacterium could play part of functions as a probiotic for pandas in a high-fiber environment, although cellulose is not a very suitable carbon source for this strain. We also demonstrated a model for understanding the dynamic organization and interactions of the various functional and regulatory networks for unicellular organisms in a high-fiber environment. As a well-characterized bacterium and a Gram-positive laboratory model, the transcriptional regulation of *B*. *subtilis* HH2 in a high-fiber environment will be a reference for other intestinal bacteria. Therefore, these results represent an important contribution to the research on the protection by the intestinal microbiota of the panda.

## Supporting Information

S1 Fig Cellulose hydrolysis halos of *B*. *subtilis* HH2. This strain has a good ability to digest cellulose; the diameter of its cellulose hydrolysis halo was 28.00±0.44 mm.
(TIF)

S1 Table GO term analysis of differentially expressed genes (DEGs). (XLSX)

S2 Table The expression levels of selected important carbon-metabolism genes. (DOCX)
