# Supplementary figures and images for: Transcriptional Regulation and Adaptation to a High-Fiber Environment in Bacillus subtilis HH2 Isolated from Feces of the Giant Panda
Source: PLoS One. 2015 Feb 6;10(2):e0116935. doi: 10.1371/journal.pone.0116935 (PMC4319723; doi:10.1371/journal.pone.0116935)

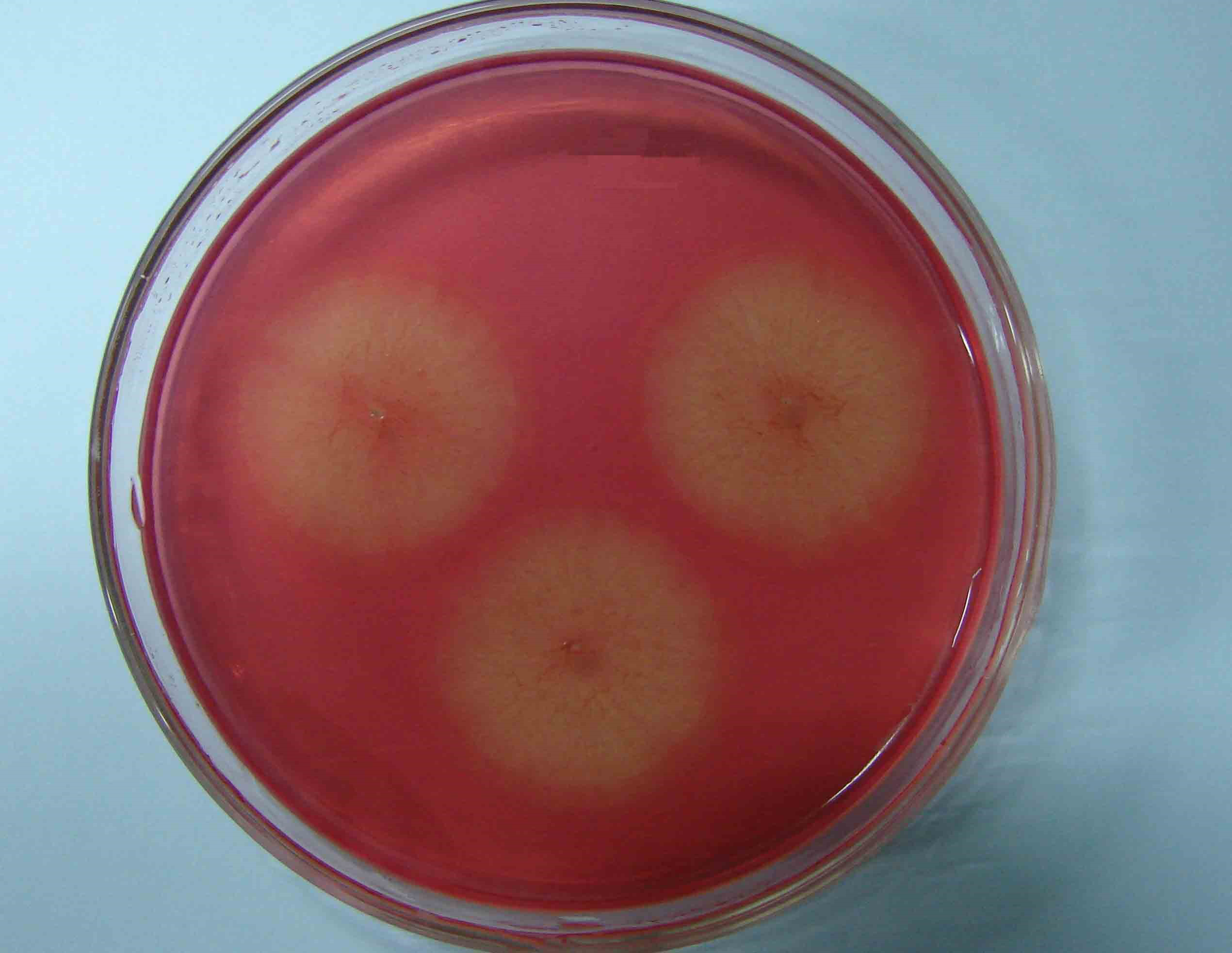

Supplement: S1 Fig — This strain has a good ability to digest cellulose; the diameter of its cellulose hydrolysis halo was 28.00±0.44 mm. (TIF) [file pone.0116935.s001.tif]
