# Supplementary material for: Transcriptional Regulation and Adaptation to a High-Fiber Environment in Bacillus subtilis HH2 Isolated from Feces of the Giant Panda
Source: PLoS One. 2015 Feb 6;10(2):e0116935. doi: 10.1371/journal.pone.0116935 (PMC4319723; doi:10.1371/journal.pone.0116935)
Supplement: S2 Table — (DOCX) [file pone.0116935.s003.docx]

Table S1 The expression level of some important genes

| Name | Cellulose  (RPKM) | Glucose  (RPKM) | Fold  （C/G） | Description |
| --- | --- | --- | --- | --- |
| U712_19480 | 626.52 | 30.14 | 20.79 | Putative 6-phospho-beta-glucosidase |
| U712_17500 | 981.95 | 55.81 | 17.59 | Pectate lyase C |
| U712_19750 | 42.43 | 4.52 | 9.39 | Beta-glucanase |
| U712_03600 | 12.94 | 6.73 | 1.92 | Putative glucosidase lpld |
| U712_19485 | 1731.58 | 62.73 | 27.60 | Lichenan-specific phosphotransferase enzyme IIA component |
| U712_19490 | 471.15 | 16.19 | 29.10 | Lichenan permease IIC component |
| U712_19495 | 943.07 | 25.43 | 37.08 | Lichenan-specific phosphotransferase enzyme IIB component |
| U712_02230 | 457.96 | 46.51 | 9.85 | Glucose starvation-inducible protein B |
| U712_17815 | 33.95 | 260.65 | 0.13 | Putative undecaprenyl-phosphate N-acetylglucosaminyl 1-phosphate transferase |
| U712_17840 | 5.44 | 86.37 | 0.06 | UDP-glucose 6-dehydrogenase tuad |
| U712_20265 | 1.2 | 25.45 | 0.05 | Gluconate operon transcriptional repressor |
| U712_20270 | 5.1 | 44.49 | 0.11 | Gluconokinase |
| U712_20275 | 3.05 | 42 | 0.07 | Gluconate permease |
| U712_20280 | 5.08 | 63.49 | 0.08 | 6-phosphogluconate dehydrogenase decarboxylating |
